# Optimal Timing of Thoracic Endovascular Aortic Repair for Late Remodeling in Acute Type B Dissection

**DOI:** 10.1016/j.atssr.2023.06.012

**Published:** 2023-07-17

**Authors:** Kentaro Kiryu, Takayuki Kadohama, Daichi Takagi, Takuya Wada, Yoshinori Itagaki, Takeshi Arai, Itaru Igarashi, Wataru Igarashi, Gembu Yamaura, Hiroshi Yamamoto

**Affiliations:** 1 Department of Cardiovascular Surgery, Akita University Graduate School of Medicine, Akita, Japan

## Abstract

**Background:**

To determine the optimal timing of thoracic endovascular aortic repair (TEVAR) for acute type B aortic dissection (TBAD), we investigated the relationship between the timing of TEVAR after onset and late aortic remodeling.

**Methods:**

Between March 2015 and August 2020, 48 patients with TBAD (39 men [81.2%]; aged 61.0 ± 10.6 years) underwent TEVAR in the acute phase (within 14 days; n = 23), subacute phase (15-90 days; n = 13), or chronic phase (>90 days; n = 12). These 3 groups were compared in terms of the true lumen (TL) area ratios, calculated by dividing the TL area by the aortic lumen area on the computed tomography image at 1 week, 1 year, 2 years, and 3 years after TEVAR.

**Results:**

At the distal-end level of the TEVAR stent, there were significant negative correlations between TEVAR timing and TL area ratio, and the cutoff value for the most effective TEVAR timing for late aortic remodeling was 14 and 8 days on the receiver operating characteristic curves with a TL area ratio of ≥80% at 1 year and 3 years after TEVAR, respectively.

**Conclusions:**

In patients with acute TBAD, acute phase TEVAR (within 14 days after onset) may be an optimal strategy to obtain sufficient aortic remodeling for a long-term postoperative period.

## Visual Abstract

**Figure:**
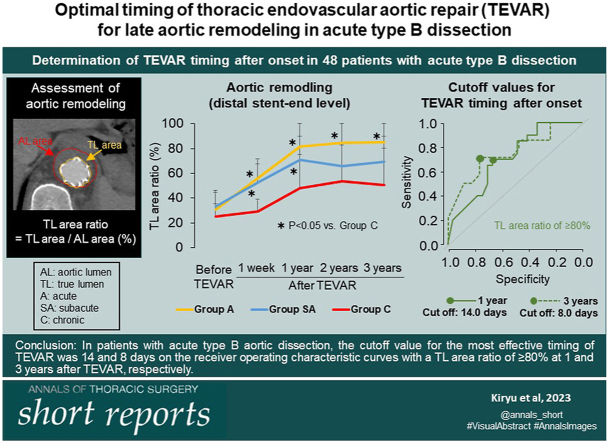

**In Short**

▪ In patients with acute type B aortic dissection, thoracic endovascular aortic repair (TEVAR) may produce a better aortic remodeling effect when it is performed in the acute or subacute phase than in the chronic phase. The optimal TEVAR timing is still unknown.
▪ Acute phase TEVAR (within 14 days after onset) may be an optimal strategy to obtain better aortic remodeling, as indicated by a true lumen/aortic lumen area ratio of ≥80% for a long-term postoperative period.

Thoracic endovascular aortic repair (TEVAR) has been demonstrated to produce a better aortic remodeling effect when it is performed in the acute (<2 weeks after onset) or subacute (2 weeks to 3 months after onset) phase than in the chronic phase (> 3 months after onset).^1^^,^^2^ The purpose of this study was to determine the exact optimal timing of TEVAR for better late aortic remodeling in patients with acute type B aortic dissection (TBAD).

## Patients and Methods

The study was approved by the institutional review board and ethics committee of the Akita University (No. 2910 on November 10, 2022). The need for individual patient consent was waived.

Between March 2015 and August 2020, 48 patients (39 men [81.2%]; aged 61.0 ± 10.6 years [40-84 years]) with TBAD (DeBakey type IIIb) underwent TEVAR. Patients with uncomplicated and complicated TBAD were enrolled because the study focused on the relationship between the timing of TEVAR and postoperative aortic remodeling, regardless of the presence or absence of TBAD-related complications. Patients were divided into 3 groups according to the timing of TEVAR: group A (23 patients [17 male], aged 60.0 ± 10.5 years), who underwent TEVAR within 14 days after onset (acute phase); group SA (13 patients [11 male], aged 59.3 ± 9.0 years), who underwent TEVAR between 15 and 90 days after onset (subacute phase); and group C (12 patients [11 male], aged 64.8 ± 11.4 years), who underwent TEVAR >90 days after onset (chronic phase). There were no significant differences between the 3 groups in terms of patients' age or sex. The duration between TBAD onset and TEVAR was 3.0 ± 3.9 days, 40.0 ± 20.6 days, and 2238 ± 2024 days in groups A, SA, and C, respectively. The indications for TEVAR are listed in the Supplemental Table.

### Surgical Procedures

The “total descending TEVAR” strategy (covered stent graft deployment to the level just above the celiac artery, in addition to entry closure) was employed in 46 patients (95.8%) and entry closure alone in 2 patients (4.2%). The proximal end of the stent graft was positioned at aortic zone 2 in 29 patients (60.4%) and aortic zone 3 in 19 patients (39.6%). All the devices used in the study were commercially available stent grafts. The provisional extension to induce complete attachment (PETTICOAT) technique^3^ was performed concomitantly with bare-metal stents in 21 patients who underwent total descending TEVAR.

### Time-Dependent Change of Aortic Remodeling During Follow-up

**Supplementary Figure 1:**
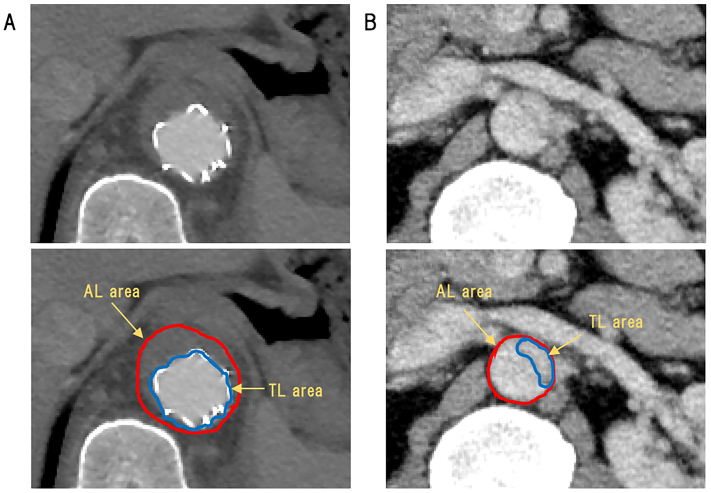


Follow-up computed tomography scanning was performed at 1 week (before discharge), 6 months postoperatively, and yearly thereafter. Aortic remodeling was assessed using a true lumen (TL) area ratio, which was calculated by dividing the TL area by the aortic lumen (AL) area (Supplemental Figure 1). For an overview of the time-dependent changes in aortic remodeling after TEVAR in the acute, subacute, or chronic phase, the TL area ratios at 1 week, 1 year, 2 years, and 3 years after TEVAR were assessed in each group at the level of the distal end of the TEVAR stent or the renal arteries.

### Relationship Between TEVAR Timing and Late Aortic Remodeling

To elucidate whether TEVAR timing after TBAD onset is related to aortic remodeling during the follow-up period, we assessed the correlation between TEVAR timing and TL area ratio at 1 year, 2 years, or 3 years after TEVAR.

### Determination of Optimal TEVAR Timing for Late Aortic Remodeling

To determine the most reliable cutoff value of TEVAR timing, we constructed receiver operating characteristic (ROC) curves for TL area ratios of ≥50%, ≥60%, ≥70%, and ≥80% at the level of the distal end of the TEVAR stent. The cutoff value was calculated by the Youden index on the ROC curve with the largest area under the curve (AUC) among them.

### Statistical Analysis

EZR statistical software was used for the statistical analysis.^4^ Continuous variables were summarized as means ± SDs of the means and compared by Student *t*-test (paired or unpaired) and *F* test (*P* < .05 was considered significant). Spearman rank correlation coefficient was used to examine the relationship between TEVAR timing and TL area ratio (*P* < .05 was considered significant). Postoperative survival and reintervention curves were constructed with the Kaplan-Meier curve.

## Results

No in-hospital deaths or TEVAR-related complications occurred after TEVAR. The AL area, TL area, and TL area ratio by study group are shown in the Table.

**Table:** Aortic Lumen Area, True Lumen Area, and True Lumen Area Ratio by Study Group

Follow-up Time	Variable	Group A	Group SA	Group C	Intergroup *P* value		
					A vs SA	A vs C	SA vs C
Before TEVAR	No. of patients	23	13	12			
	Stent end level						
	AL, mm^2^	744.4 ± 151.3	763.5 ± 205.4	1156.3 ± 350.2	.758	<.001	.003
	TL, mm^2^	226.8 ± 120.0	255.7 ± 114.7	267.5 ± 97.8	.498	.333	.793
	TL area ratio, %	30.9 ± 15.1	33.2 ± 11.6	25.0 ± 10.6	.637	.252	.089
	RA level						
	AL, mm^2^	459.0 ± 92.2	539.7 ± 185.1	854.2 ± 510.2	.099	.0013	.058
	TL, mm^2^	187.5 ± 92.4	257.2 ± 96.0	170.3 ± 58.9	.045	.573	.016
	TL area ratio, %	43.2 ± 25.3	48.4 ± 12.3	26.1 ± 14.0	.506	<.001	.042
1 week after TEVAR	No. of patients	23	13	12			
	Stent end level						
	AL, mm^2^	851.6 ± 242.7	813.1 ± 278.0	1341.0 ± 88.2	.675	<.001	<.001
	TL, mm^2^	457.8 ± 97.0	403.6 ± 102.0	365.3 ± 88.2	.134	.0011	.346
	TL area ratio, %	56.2 ± 15.2	52.4 ± 15.2	29.1 ± 10.0	.496	<.001	<.001
	RA level						
	AL, mm^2^	470.0 ± 136.8	557.5 ± 260.2	893.6 ± 571.2	.208	.002	.051
	TL, mm^2^	265.1 ± 69.3	290.8 ± 125.6	197.7 ± 85.2	.448	.019	.011
	TL area ratio, %	61.1 ± 22.7	57.1 ± 21.3	30.7 ± 24.5	.621	.001	.011
1 year after TEVAR	No. of patients	23	13	12			
	Stent end level						
	AL, mm^2^	765.3 ± 303.2	849.2 ± 323.7	1573.6 ± 937.2	.454	<.001	.019
	TL, mm^2^	574.9 ± 190.5	554.0 ± 137.5	566.5 ± 132.7	.736	.895	.825
	TL area ratio, %	81.3 ± 23.8	71.0 ± 18.8	47.8 ± 27.4	.201	<.001	.026
	RA level						
	AL, mm^2^	466.4 ± 166.4	565.6 ± 281.9	898.8 ± 655.4	.205	.006	.122
	TL, mm^2^	269.7 ± 67.5	362.0 ± 279.9	248.7 ± 136.8	.152	.559	.235
	TL area ratio, %	66.5 ± 28.2	64.7 ± 22.6	35.3 ± 23.9	.847	<.001	.019
2 years after TEVAR	No. of patients	18	11	7			
	Stent end level						
	AL, mm^2^	716.4 ± 310.3	907.6 ± 246.7	1164.2 ± 584.7	.105	.032	.266
	TL, mm^2^	558.4 ± 184.3	561.2 ± 127.8	474.4 ± 153.3	.965	.322	.253
	TL area ratio, %	84.6 ± 21.9	65.5 ± 19.0	53.8 ± 28.9	.029	.013	.361
	RA level						
	AL, mm^2^	496.9 ± 284.9	620.5 ± 276.7	983.3 ± 831.9	.167	.044	.252
	TL, mm^2^	284.9 ± 88.2	296.6 ± 82.4	209.5 ± 125.4	.750	.129	.156
	TL area ratio, %	64.9 ± 27.8	61.0 ± 28.7	34.1 ± 27.2	.746	.027	.106
3 years after TEVAR	No. of patients	15	8	5			
	Stent end level						
	AL, mm^2^	734.7 ± 378.9	861.2 ± 207.1	1168.1 ± 493.2	.411	.068	.180
	TL, mm^2^	562.4 ± 163.9	568.3 ± 113.3	479.5 ± 159.3	.931	.361	.304
	TL area ratio, %	85.2 ± 21.7	69.3 ± 20.3	50.7 ± 29.5	.118	.016	.242
	RA level						
	AL, mm^2^	509.7 ± 177.3	557.2 ± 226.4	710.0 ± 306.2	.602	.107	.363
	TL, mm^2^	286.5 ± 81.1	324.6 ± 105.2	231.7 ± 151.8	.367	.342	.256
	TL area ratio, %	62.7 ± 25.8	64.9 ± 28.6	38.1 ± 31.2	.863	.114	.180

A, underwent TEVAR within 14 days after onset (acute phase); AL, aortic lumen; C, underwent TEVAR >90 days after onset (chronic phase); RA, renal artery; SA, underwent TEVAR at 15 to 90 days after onset (subacute phase); TEVAR, thoracic endovascular aortic repair; TL, true lumen.

### Time-Dependent Change of Aortic Remodeling During Follow-up

**Figure 1:**
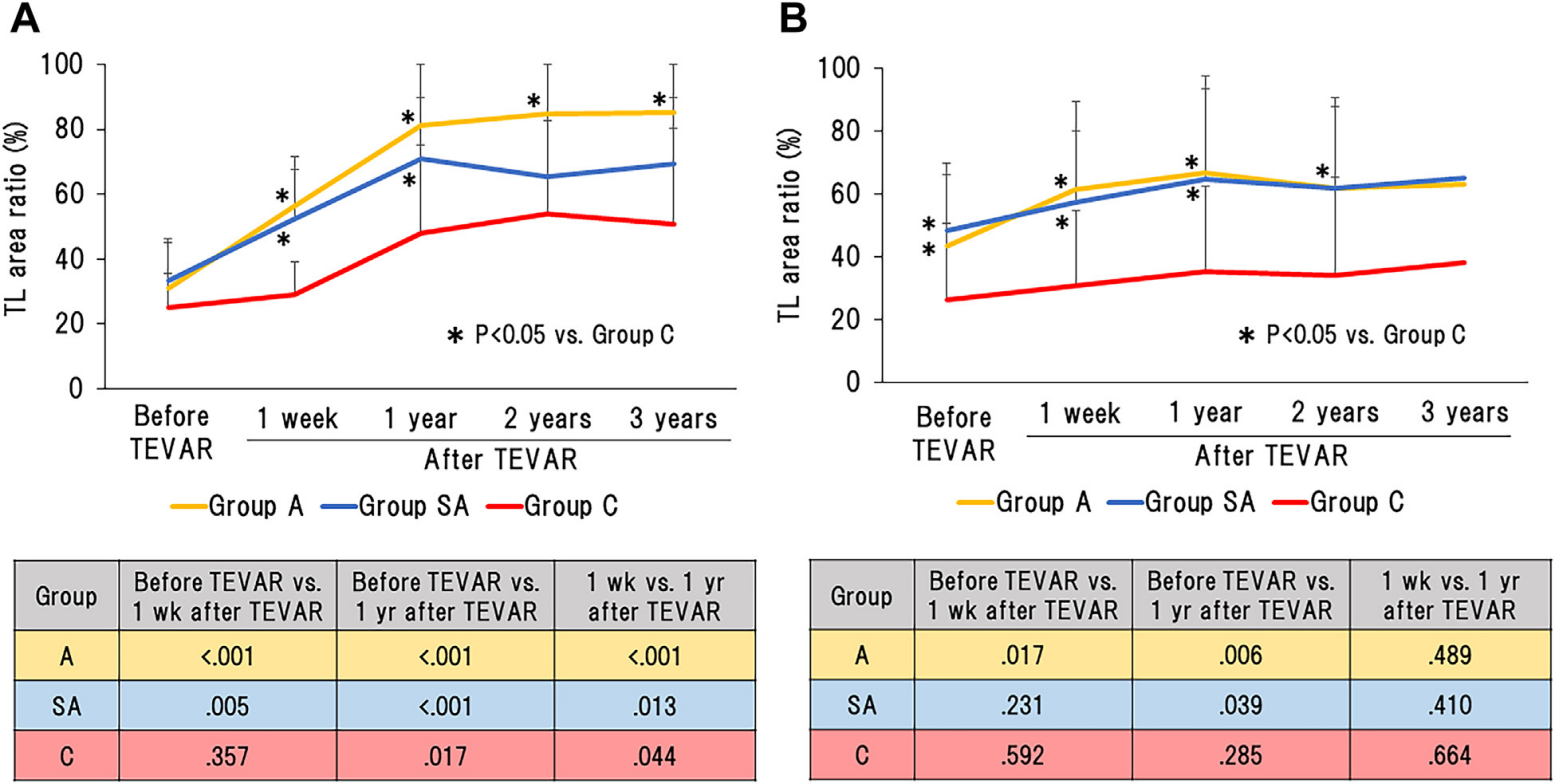
Time-dependent change of aortic remodeling during the follow-up period at the levels of (A) the distal end of the thoracic endovascular aortic repair (TEVAR) stent and (B) the renal arteries and *P* values of differences between the 3 follow-up periods (before TEVAR; 1 week after TEVAR; 1 year after TEVAR) in group A, group SA, or group C at the levels of (A, inset table) the distal end of the TEVAR stent and (B, inset table) the renal arteries. (A, underwent TEVAR within 14 days after onset (acute phase); C, underwent TEVAR >90 days after onset (chronic phase); SA, underwent TEVAR at 15 to 90 days after onset (subacute phase); TL, true lumen.)

At the level of the distal end of the TEVAR stent (Figure 1A), the TL area ratio was significantly greater in group A than in group C throughout the follow-up period, and it was significantly greater in group SA than in group C at 1 week and 1 year after TEVAR. At the renal artery level (Figure 1B), the TL area ratio was significantly greater in group A than in group C at 1 week, 1 year, and 2 years after TEVAR, and it was significantly greater in group SA than in group C at 1 week and 1 year after TEVAR.

At the level of the distal end of the TEVAR stent (inset table in Figure 1A), the TL area ratio significantly increased in groups A and SA during the first week after TEVAR and in all groups during the first year after TEVAR. At the renal artery level (inset table in Figure 1B), the TL area ratio significantly increased in group A during the first week after TEVAR and in groups A and SA during the first year after TEVAR.

### Determination of Optimal TEVAR Timing for Aortic Remodeling

**Figure 2:**
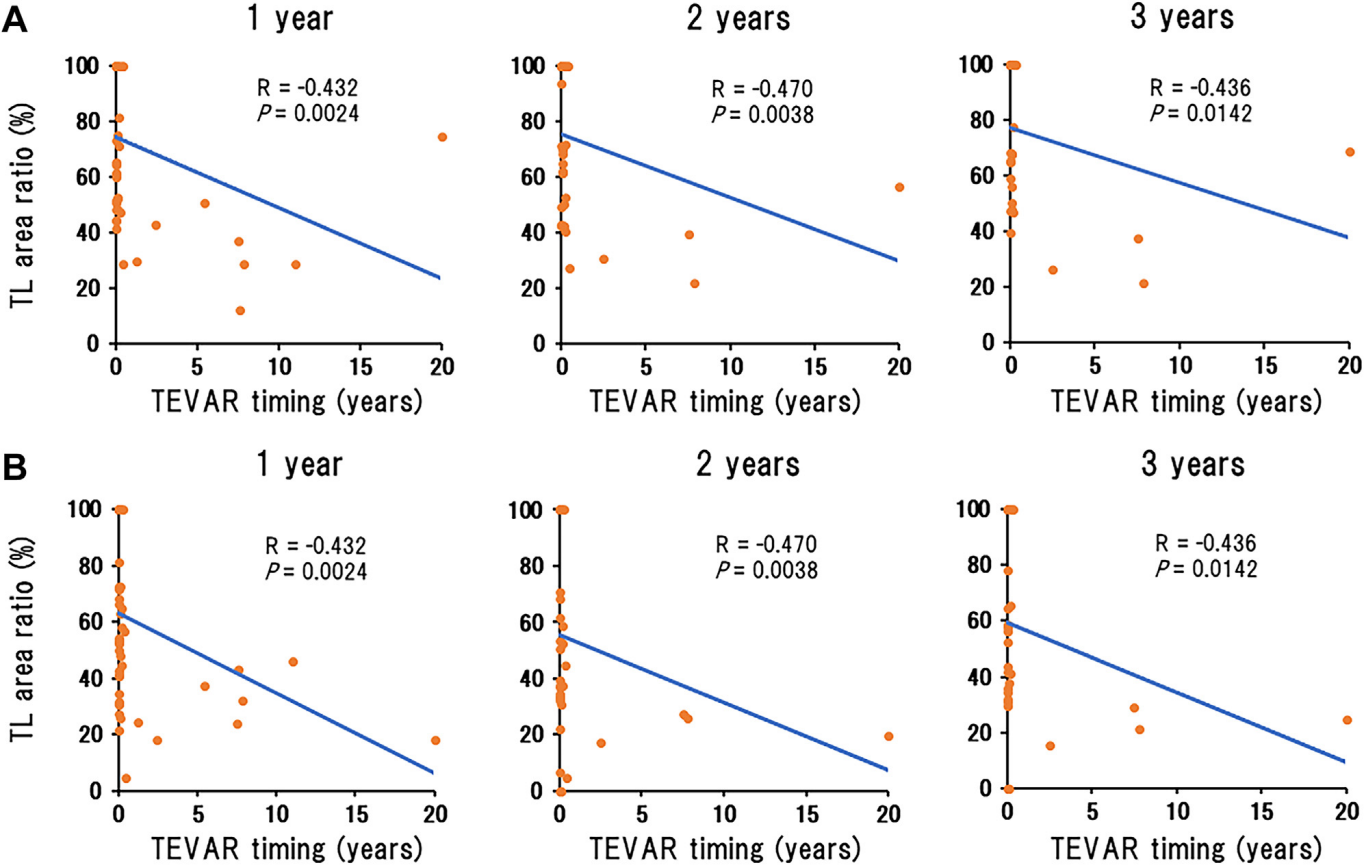
Relationship of true lumen (TL) area ratio to thoracic endovascular aortic repair (TEVAR) timing at 1 year, 2 years, and 3 years after TEVAR at the levels of (A) the distal end of the TEVAR stent and (B) the renal arteries.

**Figure 3:**
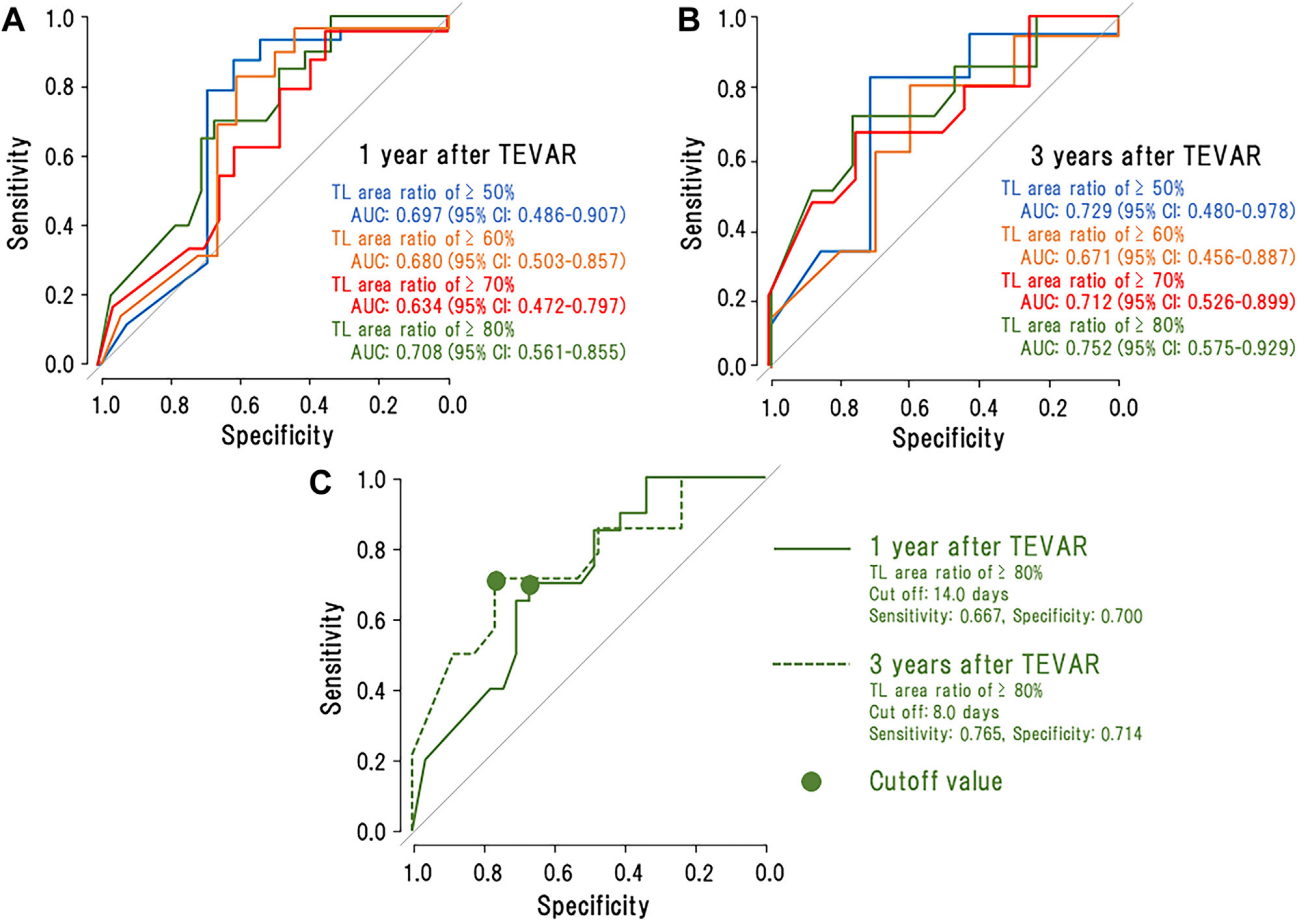
Receiver operating characteristic (ROC) curves with the true lumen (TL) area ratios of ≥50% (blue line), ≥60% (yellow line), ≥70% (red line), and ≥80% (green line) at (A) 1 year or (B) 3 years after TEVAR (thoracic endovascular aortic repair). To obtain the most reliable cutoff value (ie, TEVAR timing), the ROC curve with the largest area under the curve (AUC) was selected as the most suitable curve. At 1 year after TEVAR, the AUC was 0.697, 0.680, 0.637, and 0.708 in the ROC curves with the TL area ratio of ≥50%, ≥60%, ≥70%, and ≥80%, respectively. At 3 years after TEVAR, the AUC was 0.729, 0.671, 0.712, and 0.752 in the ROC curves with the TL area ratio of ≥50%, ≥60%, ≥70%, and ≥80%, respectively. (C) The cutoff values of the optimal TEVAR timing for aortic remodeling, indicated by closed circles on the ROC curves with the TL area ratio of ≥80% at 1 year and 3 years after TEVAR. All ROC curves depicted are constructed from the data at the level of the distal end of the TEVAR stent.

Significant negative correlations between TEVAR timing and TL area ratio were observed at 1 year, 2 years, and 3 years after TEVAR at the levels of the distal end of the TEVAR stent (Figure 2A) and the renal arteries (Figure 2B), showing that earlier TEVAR timing is associated with better aortic remodeling at both levels. To determine the reliable cutoff value, we used the ROC curves of the TL area ratio ≥80% at the level of the distal end of the TEVAR stent because the AUC was largest at 1 year (Figure 3A) and 3 years (Figure 3B) after TEVAR. The cutoff values were 14 days (sensitivity, 70.0%; specificity, 66.7%) and 8 days (sensitivity, 71.4%; specificity, 76.5%) at 1 year and 3 years after TEVAR, respectively (Figure 3C).

### Postoperative Survival and Reinterventions

**Supplementary Figure 2:**
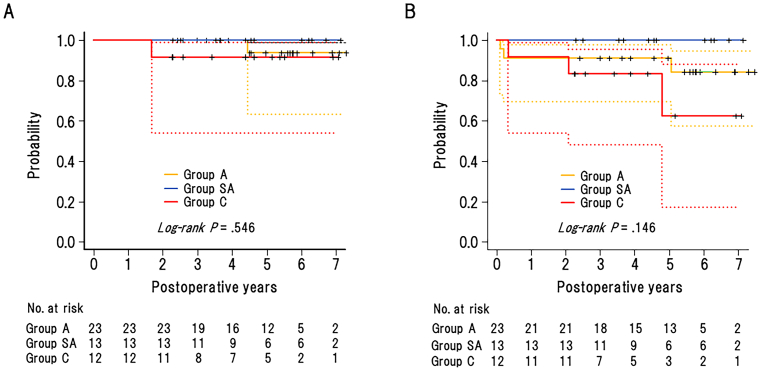

The median follow-up period was 65.1 (interquartile range [IQR], 45.0-69.8) months, 55.4 (IQR, 44.0-75.4) months, and 54.1 (IQR, 30.0-64.9) months in groups A, SA, and C, respectively. The overall survival rate at 5 years was 93.7%, 100%, and 91.6% in groups A, SA, and C, respectively (Supplemental Figure 2A). The freedom from aortic reinterventions at 5 years was 84.3%, 100%, and 62.5% in groups A, SA, and C, respectively (Supplemental Figure 2B). Distal aortic reinterventions (ie, TEVAR) were performed uneventfully in 2 patients in groups A and C (1 patient each group).

## Comment

At the distal end of the TEVAR stent, TEVAR exerted a rapid effect on the increase in the TL area ratio within approximately 1 week in the acute phase but not in the chronic phase. The mechanism responsible for the striking rapid effect of an acute phase TEVAR may be associated with less tissue stiffness of the flap during the acute phase than during the chronic phase. At the renal artery level, the TL area ratio did not increase in any group between 1 week and 1 year after TEVAR, suggesting that regardless of TEVAR timing, TEVAR is not able to exert a better remodeling effect on the downstream aortic pathologic change.

Desai and colleagues^5^ reported that in a study dealing with safe TEVAR timing in acute or subacute TBAD, a higher prevalence of in-hospital deaths, paralysis, stroke, renal failure, or retrograde type A dissection was observed in the acute phase TEVAR than in the subacute phase TEVAR. Therefore, from this study, TEVAR timing should be carefully selected as there may be a narrow range in the safe and effective period (eg, from 1 to 2 weeks after onset) even in patients with uncomplicated acute TBAD.

### Limitations

This was a single-center retrospective observational study, and the patients were not randomized. A PETTICOAT technique was inevitably added for complicated TBAD, mainly in the acute phase TEVAR group, which could introduce a sampling bias and influence the results.

### Conclusion

In patients with acute TBAD, acute phase TEVAR (within 14 days after onset) may be an optimal strategy to obtain better aortic remodeling, as indicated by a TL area ratio of ≥80% for a long-term postoperative period.

