# Supplementary material for: Optimal Timing of Thoracic Endovascular Aortic Repair for Late Remodeling in Acute Type B Dissection
Source: Ann Thorac Surg Short Rep. 2023 Jul 17;1(4):604–9. doi: 10.1016/j.atssr.2023.06.012 (PMC11708470; doi:10.1016/j.atssr.2023.06.012)
Supplement: Supplementary Table [file mmc1.docx]

Supplemental Table. Indications for TEVAR by study group

| Indications | Group A  n=23 | Group SA  n=13 | Group C  n=12 | *P value* |
| --- | --- | --- | --- | --- |
| Aortic rupture, n (%)  Lower limb ischemia, n (%)  Spinal cord ischemia, n (%)  Intestinal ischemia, n (%)  True lumen stenosis, n (%)  Retrograde type A aortic dissection, n (%)  Secondary aortic dissection or ULP, n (%)  Aortic enlargement, n (%)  Preemptive TEVAR, n (%) | 4 (7.3)  5 (21.7)  1 (4.3)  2 (8.6)  7 (30.4)  3 (13.0)  1 (4.3)  0 (0.0)  0 (0.0) | 0 (0.0)  1 (7.6)  0 (0.0)  0 (0.0)  2 (15.3)  1 (7.6)  2 (15.3)  4 (30.7)  3 (23.0) | 0 (0.0)  0 (0.0)  0 (0.0)  0 (0.0)  1 (8.3)  0 (0.0)  0 (0.0)  11 (91.6)  0 (0.0) | .0981  .157  .581  .329  .272  .421  .255  <.001  .0147 |

TEVAR, thoracic endovascular aortic repair; ULP, ulcer like projection. Categorical variables were summarized as number and percentage frequencies and compared using the Kruskal-Wallis test.
