# Supplementary material for: Optimal Timing of Thoracic Endovascular Aortic Repair for Late Remodeling in Acute Type B Dissection
Source: Ann Thorac Surg Short Rep. 2023 Jul 17;1(4):604–9. doi: 10.1016/j.atssr.2023.06.012 (PMC11708470; doi:10.1016/j.atssr.2023.06.012)
Supplement: Supplementary Figure Legends [file mmc2.docx]

**Supplemental figure legends**

**Supplemental** **Figure 1**

Measurements of the AL and TL area at the levels of the distal end of the TEVAR stent (A) and the renal arteries (B).

AL, aortic lumen; TEVAR, thoracic endovascular aortic repair; TL, true lumen.

**Supplemental Figure 2.** Kaplan–Meier curves showing overall survival (A) and freedom from re-intervention (B) during the postoperative period. Broken lines represent 95% confidence intervals (CI).
